# Comparison of the 2022 and 2017 European LeukemiaNet risk classifications in a real-life cohort of the PETHEMA group

**DOI:** 10.1038/s41408-023-00835-5

**Published:** 2023-05-12

**Authors:** Claudia Sargas, Rosa Ayala, María J. Larráyoz, María C. Chillón, Eduardo Rodriguez-Arboli, Cristina Bilbao, Esther Prados de la Torre, David Martínez-Cuadrón, Rebeca Rodríguez-Veiga, Blanca Boluda, Cristina Gil, Teresa Bernal, Juan Bergua, Lorenzo Algarra, Mar Tormo, Pilar Martínez-Sánchez, Elena Soria, Josefina Serrano, Juan M. Alonso-Dominguez, Raimundo García, María Luz Amigo, Pilar Herrera-Puente, María J. Sayas, Esperanza Lavilla-Rubira, Joaquín Martínez-López, María J. Calasanz, Ramón García-Sanz, José A. Pérez-Simón, María T. Gómez Casares, Joaquín Sánchez-García, Eva Barragán, Pau Montesinos, PETHEMA cooperative study group, Esther Prados de la Torre

**Affiliations:** 1 Grupo Acreditado de Investigación en Hematología, Instituto de Investigación Sanitaria La Fe (IIS La Fe), Valencia, Spain; 2 Hospital Universitario 12 de Octubre, CNIO, Complutense University, Madrid, Spain; 3 CIMA LAB Diagnostics-Universidad de Navarra, Pamplona, Spain; 4 Servicio de Hematología, Hospital Universitario de Salamanca (HUS/IBSAL), CIBERONC, Centro de Investigación del Cáncer-IBMCC (USAL-CSIC), Salamanca, Spain; 5 Hospital Universitario Virgen del Rocío, Instituto de Biomedicina (IBIS/CSIC), Universidad de Sevilla, Sevilla, Spain; 6 Hospital Universitario de Gran Canaria Dr. Negrín, Las Palmas de Gran Canaria, Spain; 7 IMIBIC, Hematology, Hospital Universitario Reina Sofía, UCO, Córdoba, Spain; 8 Servicio de Hematología, Grupo Acreditado de Investigación en Hematología, Hospital Universitario y Politécnico La Fe, Instituto de Investigación Sanitaria La Fe (IIS La Fe), Valencia, Spain; 9 Centro de Investigación Biomédica en Red de Cáncer (CIBERONC), Instituto de Investigación Sanitaria La Fe (IIS La Fe), Valencia, Spain; 10 Hospital General Universitario de Alicante, Alicante, Spain; 11 Hospital Universitario Central de Asturias, Instituto Universitario (IUOPA), Instituto de investigación del Principado de Asturias (ISPA), Oviedo, Spain; 12 Hospital Universitario San Pedro de Alcántara, Cáceres, Spain; 13 Hospital Universitario General de Albacete, Albacete, Spain; 14 Hospital Clínico Universitario-INCLIVA, Valencia, Spain; 15 Hospital Universitario Fundación Jiménez Díaz, Madrid, Spain; 16 Hospital Universitari General de Castelló, Castellón, Spain; 17 Hospital Universitario Morales Messeguer, Murcia, Spain; 18 Hospital Universitario Ramón y Cajal, Madrid, Spain; 19 Hospital Universitari Dr. Peset, Valencia, Spain; 20 Hospital Universitario Lucus Augusti, Lugo, Spain; 21 Servicio Análisis Clínicos, Grupo Acreditado de Investigación en Hematología, Hospital Universitario y Politécnico La Fe, Instituto de Investigación Sanitaria La Fe (IIS La Fe), Valencia, Spain; 22 Fundacion PETHEMA, Madrid, Spain

**Keywords:** Acute myeloid leukaemia, Cancer genetics, Genetic testing

## Abstract

Next-Generation Sequencing is needed for the accurate genetic risk stratification of acute myeloid leukemia according to European LeukemiaNet (ELN) guidelines. We validated and compared the 2022 ELN risk classification in a real-life cohort of 546 intensively and 379 non-intensively treated patients. Among fit patients, those aged ≥65 years old showed worse OS than younger regardless risk classification. Compared with the 2017 classification, 14.5% of fit patients changed the risk with the 2022 classification, increasing the high-risk group from 44.3% to 51.8%. 3.7% and 0.9% *FLT3*-ITD mutated patients were removed from the favorable and adverse 2017 categories respectively to 2022 intermediate risk group. We suggest that midostaurin therapy could be a predictor for 3 years OS (85.2% with vs. 54.8% without midostaurin, *P* = 0.04). Forty-seven (8.6%) patients from the 2017 intermediate group were assigned to the 2022 adverse-risk group as they harbored myelodysplasia (MDS)-related mutations. Patients with one MDS-related mutation did not reach median OS, while patients with ≥2 mutations had 13.6 months median OS (*P* = 0.002). Patients with *TP53* ± complex karyotype or inv(3) had a dismal prognosis (7.1 months median OS). We validate the prognostic utility of the 2022 ELN classification in a real-life setting providing supportive evidences to improve risk stratification guidelines.

## Introduction

The application of Next-Generation Sequencing (NGS) has increased the number of relevant molecular alterations for the management of acute myeloid leukemia (AML) [1]. This progress has substantially modified the diagnostic and prognostic classifications of AML, becoming molecular and cytogenetic alterations essential to properly diagnose and classify patients according to international guidelines [2–4].

In 2022, an updated version of the European LeukemiaNet (ELN) recommendations for diagnosis and management of AML was published [4]. The ELN genetic risk classification was revised to include additional cytogenetic and molecular markers besides measurable residual disease assessment to refine individual risk assignment [5]. One of the most important changes was the elimination of *FLT3*-ITD allelic ratio in the risk stratification; therefore, all patients with *FLT3*-ITD are now categorized as intermediate-risk irrespective of allelic ratio and concurrent *NPM1* mutation. Other major modification was the categorization of AML with myelodysplasia-related gene (MDS) mutations (*ASXL1*, *BCOR*, *EZH2*, *RUNX1*, *SF3B1*, *SRSF2*, *STAG2*, *U2AF1*, and *ZRSR2*) as adverse genetic risk. In addition, the 2017 ELN risk classification only considered biallelic mutated *CEBPA* as favorable genetic abnormality; however recent studies [6–8] have shown that only in-frame mutations affecting the basic leucine zipper (bZIP) domain of *CEBPA* confer favorable outcome. Consequently, bZIP in-frame *CEBPA* mutations (*CEBPA* bZIP) are now categorized within the favorable-risk category irrespective of their occurrence as biallelic or monoallelic mutations. Regarding cytogenetics, additional abnormalities have been included as adverse-risk factors including t(3q26.2;v) involving the *MECOM* gene or t(8;16)(p11.2;p13.3) associated with *KAT6A::CREBBP* gene fusion [9, 10]. Furthermore, hyperdiploid karyotypes with multiple trisomies (or polysomies) without structural abnormalities are not considered complex karyotypes (CK). Finally, adverse chromosomal abnormalities define poor outcome irrespective of *NPM1* mutations [11]. Although the new 2022 ELN risk stratification could refine and improve the former 2017 ELN classification, this should be confirmed in large AML series with complete NGS and cytogenetic datasets. Furthermore, validation of the 2022 ELN prognostic impact in a real-life cohort could be helpful to support its use in the routine clinical practice.

This study aims to compare and validate the 2022 ELN and 2017 ELN risk classifications in a large real-life series of newly diagnosed AML patients included in the *Programa Español de Tratamientos en Hematología* (PETHEMA) registry.

## Methods

### Patients and inclusion criteria

Since October 2017, bone marrow samples of 2434 patients with diagnosis of AML (as per WHO 2016 criteria) were analyzed in the PETHEMA central laboratories (PLATAFO-LMA project). Pediatric patients (<18 years) and acute promyelocytic leukemias were excluded, and all eligible patients were registered regardless of the treatment received. Secondary AML (sAML) was defined as follows: (1) AML after myelodysplastic syndrome and/or myeloproliferative neoplasm, or (2) AML therapy-related, or (3) AML after neoplasm not treated with radiotherapy or chemotherapy [12]. The Institutional Ethics Committee for Clinical Research of each institution approved this study. Written informed consent in accordance with the recommendations of the Declaration of Human Rights, the Conference of Helsinki, and institutional regulations were obtained from all patients.

### Genetic analysis

Molecular analyses were performed by NGS following harmonized criteria previously established by the PETHEMA group in 7 central laboratories [13]. NGS panel included 32 genes: *ASXL1, BCOR, BRAF, CALR, CBL, CEBPA, CSF3R, DNMT3A, ETV6, EZH2, FLT3, GATA2, HRAS, IDH1, IDH2, JAK2, KIT, KRAS, MPL, NPM1, NRAS, PTPN11, RUNX1, SETBP1, SF3B1, SRSF2, STAG2, TET2, TP53, U2AF1, WT1*, and *ZRSR2*. Quality parameter criteria: uniformity (>85%) and mean read depth of 1000X. Consensus criteria for variant report: all pathogenic or probably damaging variants with VAF ≥ 5% in AML key genes were reported. For variants with 1–5% VAF, only those described in hotspot regions of clinically relevant genes were considered. Cytogenetic analyses were performed locally.

### Statistics

All statistics were performed using SPSS version 22 (IBM, Armonk, NY, USA) and GraphPad Prism 4 (GraphPad, La Jolla, CA, USA) software programs. Chi square test was used to assess associations between categorical variables. Kruskal–Wallis test was used to compare the distribution of continuous variables among groups. Survival analyses were performed using the Kaplan–Meier method and the log-rank test. Cox proportional-hazards model was used to evaluate the risk of death among groups. Patients were censored at the last date they were known to be alive. *P*-value (*P*) < 0.05 was considered as statistically significant test. All *P* values reported are 2-sided.

## Results

Based on the full availability of clinical, cytogenetic and mutational data of the real-life PETHEMA cohort, 546 intensively treated patients were considered to ELN risk assessment classification (table S1). A separate analysis was conducted in 379 non-intensively treated patients according to the ELN guidelines. Median follow-up time for the global cohort was 25.3 months. Therapeutic approaches and proportion of patients who received stem cell transplant are described in tables S5 and S2, respectively.

### 2017 and 2022 ELN risk groups in intensively treated patients

**Fig. 1:**
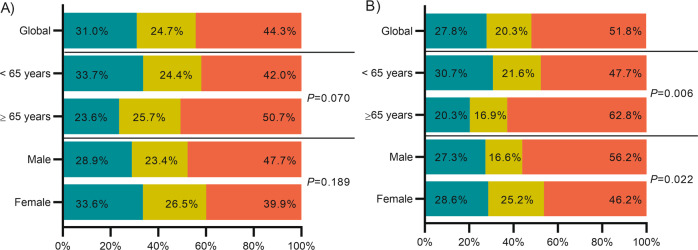
Risk categories distribution in the global cohort and according age and sex. **A** 2017 ELN and **B** 2022 ELN. Green section: Favorable risk category; Yellow section: intermediate risk category and Red section: Adverse risk category. *N* = 546 intensively treated patients.

According to 2017 ELN, 31.0% of patients were assigned to the favorable, 24.7% to the intermediate and 44.3% to the adverse-risk category. Although no significant differences were observed in risk distribution according to age, elderly patients (≥65 years) were mostly classified in the adverse risk group: <65 years: Favorable 33.7%, Intermediate 24.4% and adverse 42.0%; ≥65 years: Favorable 23.6%, Intermediate 25.7% and adverse 50.7% (*P* = 0.07). We did not find statistically significant differences in the risk group stratification according sex (Fig. 1A).

Regarding 2022 ELN risk stratification, fewer patients were classified in the favorable (27.8%) and intermediate (20.3%) risk groups, while there was an increase of adverse-risk patients (51.8%). Patients from adverse-risk category had lower leukocyte count (*P* < 0.001) and lower percentage of BM blast (*P* = 0.011) than patients in the favorable and intermediate categories (Table S2).

2022 ELN risk stratification was significantly different between younger and elderly AML patients. In younger patients, favorable and intermediate risk groups were overrepresented while adverse risk group was predominantly in elderly AML: < 65 vs. ≥65: Favorable: 30.7%–20.3%, intermediate: 21.6%–16.9%, and adverse: 47.7%–62.8% (*P* = 0.006). When comparing risk stratification according sex: 56.2% of male patients were classified in the adverse-risk category compared to 46.2% of women (*P* = 0.02). In contrast, 25.2% of women were classified in the intermediate category vs. 16.6% of male patients. Similar distribution was observed in the favorable-risk category (27.3–28.6%; Fig. 1B).

Two patients (0.4%) classified according to the 2017 ELN risk stratification showed molecular alterations that allowed them to be classified in more than one risk group. One patient had mutated *NPM1* and *FLT3*-ITD (high ratio) with mutated *RUNX1* and it was classified in the intermediate risk group. Another patient with *FLT3*-ITD (high ratio) + WT-*NPM1* and biallelic *CEBPA* mutations was assigned to the favorable risk group (Table S3).

We detected a slight increase in the percentage of patients with an ambiguous classification according to 2022 ELN (*N* = 10, 1.8%) (Table S4). Nine patients with adverse-risk cytogenetic alterations and *FLT3*-ITD with WT-*NPM1* were assigned to the adverse risk group. Similarly, due to the recognition of AML with *CEBPA* bZIP mutations as a biological entity with favorable prognosis, one patient with a *CEBPA* bZIP mutation and *FLT3*-ITD was assigned to the favorable risk group.

### Outcomes according to 2017 and 2022 ELN risk in intensively treated patients

**Fig. 2:**
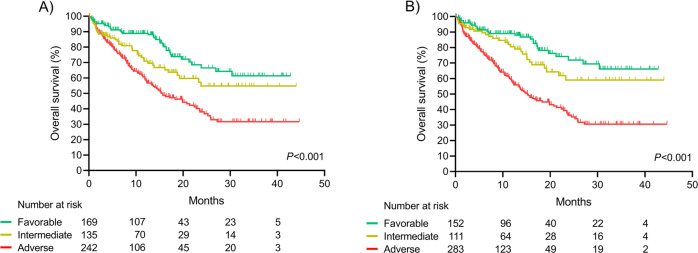
Outcomes of patients according to ELN risk categories. **A** 2017 ELN and **B** 2022 ELN.

According to 2017 ELN risk stratification, median overall survival (OS) for the whole cohort was not reached in the favorable and intermediate risk groups; while in the adverse risk group median OS was 15.7 months (95%CI 11.3–20.1; *P* < 0.001; Fig. 2A). Regardless of risk group, we detected a significantly lower median OS in patients aged ≥65 years old when compared to younger patients (Fig. S1A, B).

For the global cohort, the risk of death of intermediate and adverse-risk patients was 1.7 (95%CI 1.1–2.6; *P* = 0.02) and 2.7 (95%CI 1.9–4.0; *P* < 0.001) relative to favorable-risk group. Specific OS and risk of death of young and elderly AML patients according 2017 ELN is described in supplementary material (Figs. S2 and S5A).

According to 2022 ELN risk, median OS was not reached in the favorable and intermediate risk groups. Median OS in the adverse risk group was 15.2 months (95%CI 11.8–18.6; *P* < 0.001; Fig. 2B). Overall, intermediate risk group did not show a significant increased risk of death relative to favorable risk group [1.5 (95%CI 0.9–2.6; *P* = 0.111)]. Adverse-risk patients had an increased risk of death of 3.5 (95%CI 2.2–5.0; *P* < 0.001; Fig. S5B).

Specific OS and risk of death of young and elderly AML patients according to 2022 ELN is described in supplementary material, Figs. S3, S4, and S5B).

### 2017 and 2022 ELN in non-intensively treated patients

Among 379 non-intensively treated patients, 2017 ELN risk distribution was 18.2% favorable, 19.8% intermediate and 62% adverse. The median OS was 9.4 (95%CI 5.5–13.5), 11.5 (95%CI 5.6–17.4), and 6.5 (95%CI 4.8–8.2) months for favorable, intermediate and adverse-risk groups respectively (*P* = 0.016; Fig. S6A).

According to 2022 ELN risk distribution for non-intensively treated patients was 16.1% favorable, 11.9% intermediate and 72% adverse. Poor OS was observed for all risk categories: median OS favorable: 10.9 (95%CI 4.9–16.9), intermediate: 8.3 (95%CI 2.3–14.3), and adverse: 7.1 (95%CI 5.2–8.9; *P* = 0.219; Fig. S6B).

### Comparison of risk category assignment between 2017 and 2022 ELN criteria

**Fig. 3:**
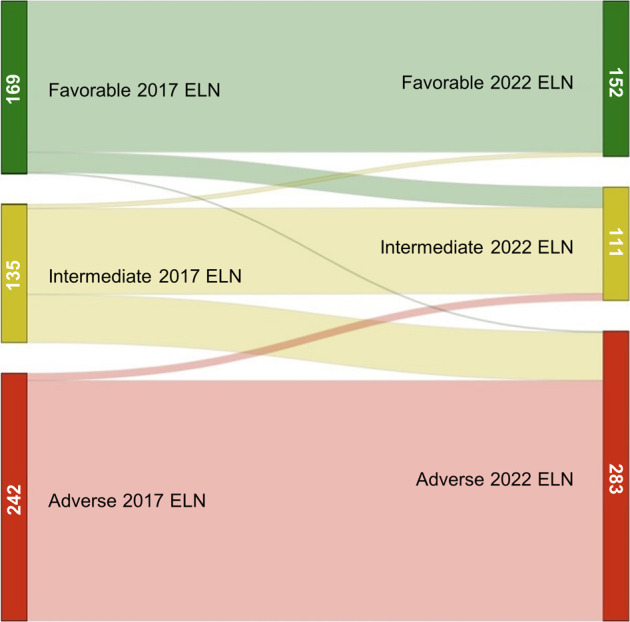
Sankey diagram comparing 2017 ELN and 2022 ELN risk categories.

Among intensively treated patients, 79 patients (14.5%) were classified into different risk groups in each classification (Fig. 3). Most transitions (12.5%) involved assignment to a worse prognosis group: 20 patients (3.7%) transitioned from the 2017 ELN favorable group to the 2022 ELN intermediate group since *FLT3*-ITD allelic ratio was not considered for risk assessment. Forty-seven (8.6%) intermediate risk patients according to 2017 ELN were assigned to an adverse risk group in the 2022 ELN classification as they harbored mutations in MDS-related genes. Only one patient (0.2%) with double *CEBPA* mutations transitioned from favorable 2017 ELN risk group to an adverse 2022 ELN as no mutation affected the bZIP domain and MDS related gene mutations were detected. On the other hand, transitions from adverse to intermediate risk group were mostly due to the consideration of *FLT3*-ITD mutations as intermediate-risk despite of high allelic ratio (*N* = 5, 0.9%). Finally, four patients (0.7%) harbored *CEBPA* bZIP mutations as the only clinically relevant alteration and transitioned from 2017 ELN intermediate category to the favorable group according to 2022 ELN classification (Table 1).

**Table 1:** Patients with different risk classification according to 2017 ELN and 2022 ELN.

N	(%)	Molecular features	2017 ELN risk classification	2022 ELN risk classification
20	3.7	Mutated *NPM1* with low allelic ratio *FLT3*-ITD	Favorable	Intermediate
1	0.2	Biallellic mutated *CEBPA* (not bZIP domain) + mutated MDS genes	Favorable	Adverse
4	0.7	bZIP in frame mutated *CEBPA* (only one *CEBPA* mutation)	Intermediate	Favorable
47	8.6	Mutated MDS genes (not *RUNX1* and *ASXL1)*	Intermediate	Adverse
4	0.7	High allelic ratio *FLT3*-ITD	Adverse	Intermediate
1	0.2	Hyperdiploid karyotype + high allelic ratio *FLT3*-ITD	Adverse	Intermediate
2	0.4	Hyperdiploid karyotype	Adverse	Intermediate

Outcomes of reclassified patients supported 2022 ELN modifications: new favorable patients harboring *CEBPA* bZIP mutations, showed good prognosis with no reported deaths at 31 months (Fig. S7A). Furthermore, outcome of new intermediate risk patients (21.8 months; 95%CI not reached) was similar to those already classified as intermediate risk patients (*P* = 0.679; Fig. S7B). Finally, new adverse risk patients showed a similar prognosis (13.7 months; 95%CI 7.0–20.5) to those previously classified in the adverse risk category (*P* = 0.794; Fig. S7C). Comparison of the reclassified patients with their previous risk-group also supports 2022 ELN reclassification although the small sample size limits to reach statistically significant results in some subgroups (Fig. S8).

### Survival in genetic subgroups of the 2022 ELN risk categories

No differences in OS were detected among favorable risk genetic subgroups [*NPM1*mut without *FLT3*-ITD, *CEBPA* bZIP, inv(16) and t(8;21); *P* = 0.741] (Table 2 and Fig. S9A). Patients with these mutations showed 2-years OS rates between 61% and 75%.

**Table 2:** Outcomes according to genetic subsets within the 2022 ELN.

Category	N	1-year OS (%)	2-year OS (%)	3-year OS (%)	*P* value
Favorable					
* NPM1*mut, *FLT3*-ITD WT	99	86.9	75.1	66.4	0.741
* CEBPA-*bZIP	9	100.0	75.0	–	
inv(16)	25	91.8	68.5	68.5	
*t*(8;21)	17	81.4	61.0	61.0	
Intermediate					
* NPM1*mut, *FLT3*-ITD mut	45	74.7	47.9	47.9	0.201
* NPM1* WT, *FLT3*-ITD mut	11	74.1	74.1	74.1	
Other abnormalities	44	88.6	72.7	72.7	
t(9;11)	1	–	–	–	
Adverse					
inv(3)	10	15.6	–	–	0.002
(−5, −7, −17)	35	56.8	34.4	23.0	
Complex karyotype and *TP53*	28	22.4	22.4	–	
Mutated *TP53*	5	45.0	–	–	
Complex karyotype	27	57.7	25.6	25.6	
MDS-mutated genes	145	59.5	38.2	38.2	
*t*(X;11)	12	54.1	27.0	–	
*t*(6;9)	5	100.0	–	–	
*t*(9;22)	3	–	–	–	

Analysis for the genetic subsets within the intermediate risk group (*NPM1*mut with *FLT3*-ITD, *NPM1*-WT with *FLT3*-ITD, t(9;11) and other cytogenetic and molecular abnormalities), did not show significant differences in OS among subgroups (*P* = 0.201; Table 2 and Fig. S9B). Twenty-two patients with *FLT3* mutations from the intermediate risk group were treated with midostaurin-based regimens, resulting in a significant (*P* = 0.042) better outcome than those treated with standard therapy with 3-years OS rates of 85.2% and 54.8%, respectively (Fig. S10).

We found significant distinct outcomes (*P* = 0.002) among adverse genetic abnormalities [inv(3), (−5, −7, −17), CK, mutated *TP53*, CK + *TP53*, MDS-mutated genes, t(X;11), t(6;9) and t(9;22)]. Patients with inv(3), mutated *TP53* and CK + *TP53* showed the worst median OS: 8.3 months (95%CI 0–16.8), 7.1 months (95%CI 0–15.0), and 3.5 (95%CI 0.8–6.2), respectively (Table 2 and Fig. S9C). Patients harboring t(6;9) (*N* = 5) or t(9;22) (*N* = 3) were excluded from the analysis because of the small sample size.

**Fig. 4:**
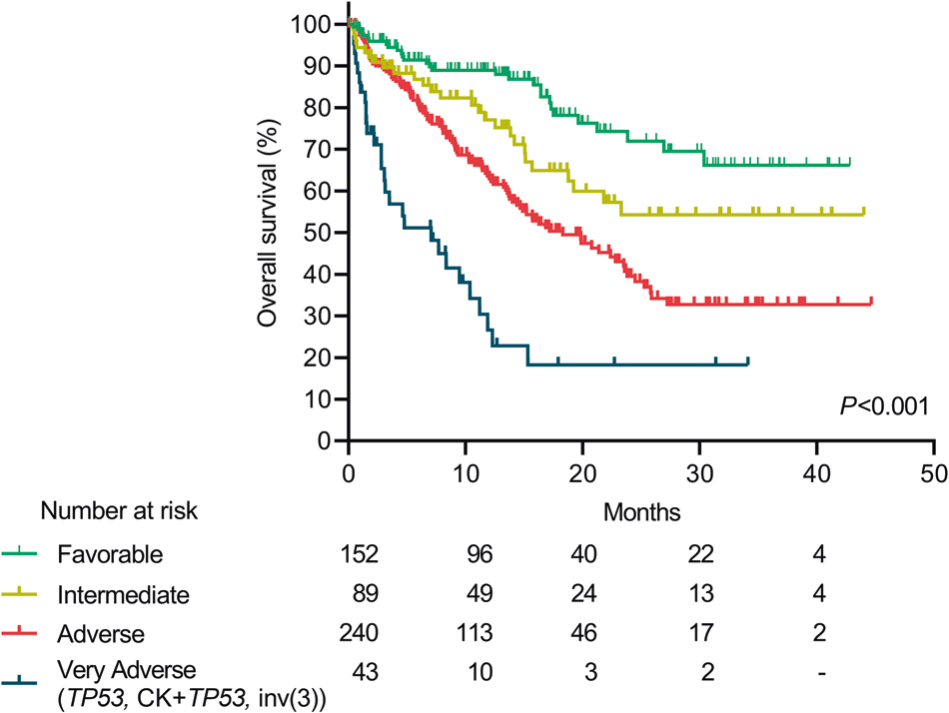
Outcomes of patients according to the proposed refinement of the 2022 ELN risk categories.

When grouped together, patients harboring inv(3), mutated *TP53* or CK + *TP53* genetic abnormalities showed a worse median OS (7.1 months; 95%CI 1.4–12.8) as compared to other adverse risk genetic groups (*P* < 0.001; Fig. S11). Together inv(3), mutated *TP53* or CK + *TP53* (“very adverse risk group”) had a higher risk of death than “adverse-risk group” in 2022 ELN [2.5 (95%CI 1.7–3.9; *P* < 0.001)] (Figs. 4 and S12).

**Fig. 5:**
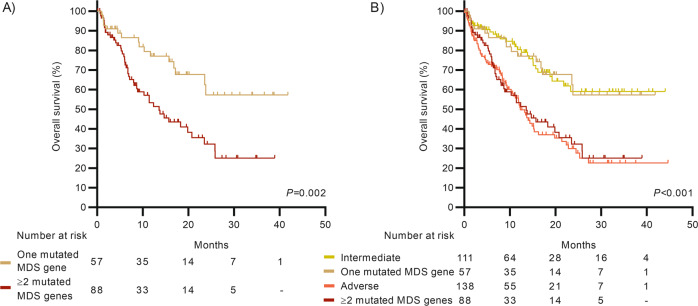
Outcomes of MDS mutated patients. **A** According to the number of mutated genes and **B** regarding intermediate and adverse 2022 ELN risk groups. MDS myelodysplasia-related genes.

MDS-related genes as per 2022 ELN risk stratification had an adverse outcome with a median OS of 19.9 months (95%CI 13.1–26.6). However, patients with only one mutated MDS gene did not reach median OS while patients with two or more mutated genes showed a median OS of 13.6 months (95%CI 9.0–18.1; *P* = 0.002; Fig. 5A). Furthermore, OS in patients with one MDS-mutated gene was similar to patients classified in the 2022 ELN intermediate group (3-year OS rate of 57.6% vs. 59.6%; *P* = 0.978) while patients with ≥2 MDS genes showed an OS similar to the adverse-risk group (3-year OS rate of 25.7% vs. 30.6%; *P* = 0.391; Fig. 5B).

Presence of MDS-related mutations in the favorable risk group (*N* = 28; 18.4%) did not impact OS (*P* = 0.986). Although MDS mutations were weakly represented in the intermediate group (*N* = 6; 5.4%) they conferred worse outcome [Median OS for MDS mutated patients: 14.2 months (95%CI 5.9–22.4) vs. not reached in patients without MDS mutations] (*P* = 0.011). The MDS mutated patients included in this group only harbored one MDS mutated gene, and had *NPM1* and *FLT3*-ITD mutations.

## Discussion

In this study we have compared the 2017 and 2022 ELN classifications in order to validate new modifications in a real-life cohort of patients from the PETHEMA cooperative group in a centralized laboratory network using harmonized NGS studies. We have validated the prognostic impact of 2022 ELN classification, which is able to properly discriminate among the three risk-groups, like the 2017 ELN. However, it is noteworthy that the risk of death of patients from the intermediate risk group was not significantly worse than favorable-risk in 2022 ELN, suggesting that the 2022 ELN is less effective at separating out intermediate-risk patients than the 2017 ELN. This could be due to improved OS in this group due to the removal of the MDS-type mutations to the adverse group. Furthermore, our analyses indicate that the reallocation of single MDS-mutated patients to the intermediate risk group could improve the sensitivity of the 2022 ELN classification. The main prognostic difference between both classifications is that the 2022 ELN increases the burden of the adverse risk group, leading to slightly better survival rates among the favorable and intermediate groups as compared to the 2017 ELN.

For both editions the adverse risk group was the most represented, followed by the favorable and intermediate risk groups. Similarly to Lachowiez et al. [14], our analysis showed an increase of 7.5% of patients classified in the 2022 ELN adverse risk group mainly due the association of MDS-mutations with high-risk disease. Furthermore, Papaemmanuil and others have recently described stronger enrichment of MDS mutations in secondary AML and older patients [15, 16]. This finding could explain our results in elderly patients with more than 60% of patients allocated in the adverse risk category following 2022 ELN criteria. In contrast, younger patients were more likely to belong to favorable and intermediate risk groups due to higher incidence of *NPM1* and *FLT3* mutations, which also had a significant impact in terms of eligibility for targeted therapy treatment [17]. It should be noted that our study validates 2022 ELN risk groups in a real-world setting, contrarily to the BEAT-AML clinical trial analyses [14], thus supporting broad applicability of these classifications in routine practice. Nevertheless, we should highlight that since the last update of the 2017 ELN, which included the assessment of *ASXL1*, *RUNX1*, and *TP53* mutations, the demand to perform a NGS panel at diagnosis has increased in the last 2022 ELN revision as it includes a greater number of alterations only addressable by NGS. However, NGS testing is not yet widely affordable for many patients and could harm the assessment of new clinically relevant markers in the real-world setting.

Among intensively treated patients, older age was strongly associated with a worse prognosis regardless of the ELN risk group, suggesting less applicability for elderly patients [18, 19]. This has been widely described by several cooperative group trials and population-based studies which have demonstrated that advanced age at the time of AML diagnosis is clearly associated with poor outcome [20–22]. On the other hand, the ELN risk classifications (both 2017 and 2022) might be used for clinical management of intensively treated patients. According to our results and previous studies [23] non-intensively treated patients have dismal prognosis regardless of ELN risk group.

The consideration of all *FLT3*-ITD mutations as intermediate risk markers into the 2022 ELN classification led to reallocate 3.7% of patients from the 2017-favorable to the 2022-intermediate risk and in 0.9% from the 2017-adverse to the 2022-intermediate categories. However, it is difficult to interpret the benefit of this risk-adjustment based on (1) several studies showing the impact of *FLT3*-ITD allelic ratio, and in particular a recently reported series of 2901 patients by PETHEMA supporting a cutoff of 0.5 for OS and 0.8 for relapse-free survival;[24] and (2) the impact of targeted therapy with midostaurin in patients with *FLT3*-ITD mutations. In fact, we confirm in the real-life setting that patients receiving front-line midostaurin had significant improved OS than those receiving standard regimens [23, 25].

Our results showed that patients with mutated *TP53*, CK + *TP53* or inv(3) could be grouped in an independent risk category with a very poor prognosis, being CK + *TP53* those with the worst prognosis [26, 27]. AML with mutated *TP53* has been widely recognized as a molecular subgroup with a very poor prognosis. Some authors refer to it as “the worst of any” with a particularly dismal prognosis especially when a CK is also present [28]. Our finding is consistent with the refinement of the 2017 ELN classification suggested by Herold et al., who already proposed a very adverse risk subgroup which encompasses patients with *TP53* mutations and CK [29]. Furthermore, the updated genomic AML classification of Tazi et al., considers CK/*TP53* and inv(3) as molecular markers of highly chemoresistant disease and relapse-related mortality [30]. In this regard, the research conducted by Grob et al. showed that only mutated *TP53* is determinant of “very adverse risk” AML regardless of concomitance with CK, which has been validated in our cohort. However, we must be prudent as *TP53* is a very heterogeneous entity which still needs to be well determined and our numbers were relatively small. This result contrasted with our results in the genetic subgroup of *CEBPA*-bZIP mutations which were the genetic abnormalities with best OS, in line with previous analyses [14].

MDS-related gene mutations have become significant in recent studies of the molecular basis of AML although its prognostic value lacks unanimous agreement. The last AML genomic classification [30], established a specific association with adverse outcomes for patients with two or more MDS gene mutations. However, 2022 ELN recommendations have not supported any differences in this respect. We show that up to 26% of patients will fall into the adverse risk 2022 ELN category due to MDS-gene mutations, becoming the biggest genetic subgroup now. Furthermore, the main risk group change between 2017 and 2022 ELN classifications was driven by the implementation of this new adverse risk subgroup. However, we show that patients with only one mutated MDS gene, representing roughly 40% of this category, had similar outcomes than intermediate risk group, while patients with two or more mutations had an OS similar to the remaining adverse risk group patients. Our results support the observations of Tazi et al., of lower response rate to induction chemotherapy and higher benefit after hematopoietic stem cell transplantation in patients harboring two or more MDS mutations [30]. Nonetheless, we recommend reassessing the appropriateness of classifying as adverse risk AML patients with a single mutation in one of the so called MDS-related genes (*ASXL1*, *BCOR*, *EZH2*, *RUNX1*, *SF3B1*, *SRSF2*, *STAG2*, *U2AF1*, and *ZRSR2)* and no other adverse genetic factor [31]. Furthermore, although in the intermediate risk group our results are not consistent due to the small sample size, it would be interesting to assess the impact of MDS mutations in this risk group.

Our study has some limitations: (1) to be comparable with other studies validating ELN classifications we pick-up OS as the main predicted outcome. However, we believe that genetic risk classifications should anticipate chemoresistance and/or relapse occurrence, as patients can die by treatment toxicity or other causes unrelated to leukemic biology itself; (2) although we analyze a modern series of patients, the therapeutic landscape in AML is rapidly evolving and we cannot properly analyze the impact of novel approaches in genetic risk assessment; and (3) our registry departed from 2434 patients with complete molecular data, but only 546 intensively and 379 non-intensively treated subjects had full clinical and cytogenetic data-set available and were used to assess the new 2022 ELN classification. We are working to increase the evaluable sample size and provide further insights in future analyses.

In summary, our study provides first validation of 2022 ELN risk stratification in the real-world setting. When compared with the 2017 ELN, 14.5% of patients were reclassified according to novel 2022 ELN criteria increasing the burden of the adverse risk group. Additional studies are needed to better define risk stratification among *FLT3*-ITD patients in the era of targeted inhibitors. The allocation of AML patients into the adverse risk group based on the presence of a single MDS-related gene mutation remains as another critical issue to be solved.

## Supplementary information

Supplementary Material

## Data Availability

The datasets generated during the current study are available from the corresponding author on reasonable request.
