# Supplementary material for: Comparison of the 2022 and 2017 European LeukemiaNet risk classifications in a real-life cohort of the PETHEMA group
Source: Blood Cancer J. 2023 May 12;13(1):77. doi: 10.1038/s41408-023-00835-5 (PMC10182047; doi:10.1038/s41408-023-00835-5)
Supplement: Supplementary file 1 — Supplementary Material [file 41408_2023_835_MOESM1_ESM.pdf]

Comparison of the 2022 and 2017 European LeukemiaNet risk classifications in a real-life cohort of the PETHEMA group

Claudia Sargas [1], Rosa Ayala [2], María J. Larráyoz [3], María C. Chillón [4], Eduardo Rodriguez-Arboli [5], Cristina Bilbao [6], Esther Prados de la Torre [7], David Martínez-Cuadrón [8,9], Rebeca Rodríguez-Veiga [8], Blanca Boluda Pascual [8], Cristina Gil [10], Teresa Bernal [11], Juan Bergua [12], Lorenzo Algarra [13], Mar Tormo [14], Pilar Martínez-Sánchez [2], Elena Soria [5], Josefina Serrano [7], Juan M. Alonso-Dominguez [15], Raimundo García [16], María Luz Amigo [17], Pilar Herrera-Puente [18], María J. Sayas [19], Esperanza Lavilla-Rubira [20], Joaquín Martínez-López [2], María J. Calasanz [3], Ramón García-Sanz [4], José A. Pérez-Simón [5], María T. Gómez Casares [6], Joaquín Sánchez-García [7], *Eva Barragán [9,21], *Pau Montesinos [8,9], # PETHEMA cooperative study group.

* Co-senior authors. These authors contributed equally to this work

Listed in the Appendix

1 Grupo Acreditado de Investigación en Hematología, Instituto de Investigación Sanitaria La Fe (IIS La Fe), Valencia, Spain.

2 Hospital Universitario 12 de Octubre, CNIO, Complutense University, Madrid, Spain.

3 CIMA LAB Diagnostics-Universidad de Navarra, Pamplona, Spain.

4 Servicio de Hematología, Hospital Universitario de Salamanca (HUS/IBSAL), CIBERONC, Centro de Investigación del Cáncer-IBMCC (USAL-CSIC), Salamanca, Spain.

5 Hospital Universitario Virgen del Rocío, Instituto de Biomedicina (IBIS / CSIC), Universidad de Sevilla, Sevilla, Spain.

6 Hospital Universitario de Gran Canaria Dr. Negrín, Las Palmas de Gran Canaria, Spain.

7 IMIBIC, Hematology, Hospital Universitario Reina Sofía, UCO, Córdoba, Spain.

8 Servicio de Hematología, Grupo Acreditado de Investigación en Hematología, Hospital Universitario y Politécnico La Fe, Instituto de Investigación Sanitaria La Fe (IIS La Fe), Valencia, Spain.

9 Centro de Investigación Biomédica en Red de Cáncer (CIBERONC), Instituto de Investigación Sanitaria La Fe (IIS La Fe), Valencia, Spain.

10 Hospital General Universitario de Alicante, Alicante, Spain.

11 Hospital Universitario Central de Asturias, Instituto Universitario (IUOPA), Instituto de investigación del Principado de Asturias (ISPA), Oviedo, Spain.

12 Hospital Universitario San Pedro de Alcántara, Cáceres, Spain.

13 Hospital Universitario General de Albacete, Albacete, Spain.

14 Hospital Clínico Universitario-INCLIVA, Valencia, Spain.

15 Hospital Universitario Fundación Jiménez Díaz, Madrid, Spain.

16 Hospital Universitari General de Castelló, Castellón, Spain.

17 Hospital Universitario Morales Messeguer, Murcia, Spain.

18 Hospital Universitario Ramón y Cajal, Madrid, Spain.

19 Hospital Universitari Dr. Peset, Valencia, Spain.

20 Hospital Universitario Lucus Augusti, Lugo, Spain.

21 Servicio Análisis Clínicos, Grupo Acreditado de Investigación en Hematología, Hospital Universitario y Politécnico La Fe, Instituto de Investigación Sanitaria La Fe (IIS La Fe), Valencia, Spain.

Address for correspondence:

Pau Montesinos, M.D., Ph.D.

Hematology Department (G block, 7th floor). Hospital Universitari i Politècnic La Fe.

Avinguda Fernando Abril Martorell, 106. CP. 46026 Valencia, Spain.

Tlf: +34 961 244925

e-mail: montesinos_pau@gva.es

Outcomes according to 2017 and 2022 ELN risk in intensively treated patients

According to 2017 ELN risk stratification, median OS for patients aged <65 years old, was not reached in the favorable and intermediate risk groups and it was 19.9 months (95%CI 13.2-26.5) for adverse risk (*P*<0.001) (Figure S1A). Patients aged ≥65 years old had lower median OS for all risk groups: Favorable (30.4 months, 95%CI 15.3-45.4), intermediate (16.8 months, 95%CI 7.4-26.2) and adverse (9.4 months, 95%CI 4.8-13.9) (*P*=0.008) (Figure S1B). Only elderly patients from the intermediate (*P*=0.007) and adverse (*P*=0.011) risk groups showed a significant worse OS than young patients belonging to the same risk-groups (Figure S2).

Lower median OS was also detected in patients aged ≥65 years according to 2022 ELN risk stratification: Younger patients did not reach median OS for favorable and intermediate risk groups, while adverse risk patients showed a median OS of 19.9 months (95%CI 13.4-26.3) (*P*<0.001) (Figure S3A). Patients aged ≥65 years showed lower median OS for all risk groups: Favorable (30.4 months, 95%CI Not reached), intermediate (18.8 months, 95%CI 12.5-25.1) and adverse (9.4 months, 95%CI 6.3-12.5) (Figure S3B). Only elderly patients from the adverse risk group showed worse OS than young patients (*P*=0.004) (Figure S4).

Risk of death according to 2017 and 2022 ELN risk in intensively treated patients

For 2017 ELN risk categories, young and elderly AML patients classified in the adverse risk groups, showed an increased risk of death of 2.9 (95%CI 1.8-4.6, *P*<0.001) and 2.3 (95%CI 1.2-4.3, *P*<0.001) concerning its relative favorable-risk groups. We did not detect any difference for either intermediate risk groups (<65 years: 1.6; 95%CI 0.9-2.8; *P*=0.131; ≥65 years: 1.8 (95%CI 0.9-3.7, *P*=0.113) (Figure S5A). When comparing the same risk group according to age, elderly patients classified in the intermediate and adverse risk groups showed an increased risk of death of 2.3 (95%CI 1.2-4.3, *P*=0.009) and 1.6 (95%CI 1.1-2.4, *P*=0.01) regarding to young patients.

Regarding risk of death according 2022 ELN categories, only younger and elderly patients classified in the adverse risk group, showed a statistically significant increased risk of death compared to their respective favorable-risk groups (Figure S5B). Regarding age impact, only patients ≥65 years from the adverse risk group, showed an increased risk of death compared to adverse-risk young patients [HR 1.7 (95%CI 1.2-2.4; *P*=0.004)]. No significant differences were reached were reached when comparing favorable and intermediate risk groups between young and elderly AML patients. [Favorable: HR 2.1 (95%CI 0.9-4.5; *P*=0.069); Intermediate: HR 1.8 (95%CI 0.8-4.0; *P*=0.116)]. (Figure S4)

Figures

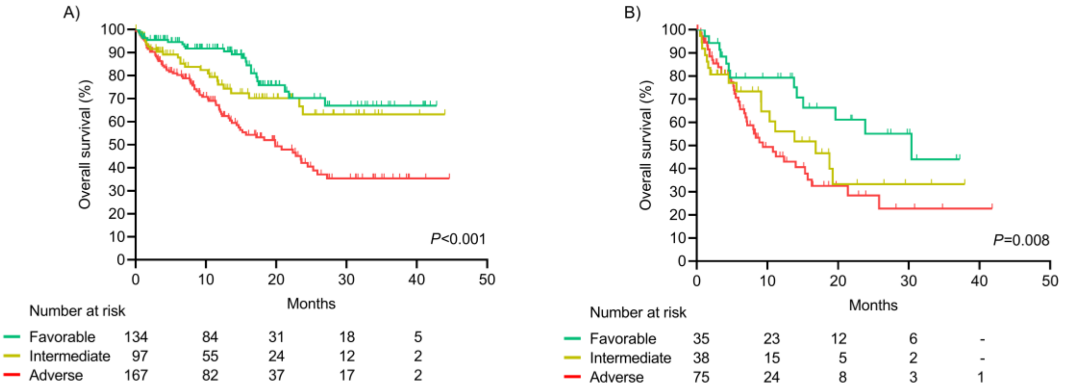

Figure S1. Overall survival after intensive therapy according to 2017 ELN risk for: A) young patients (<65 years) and B) elderly patients (≥65 years)

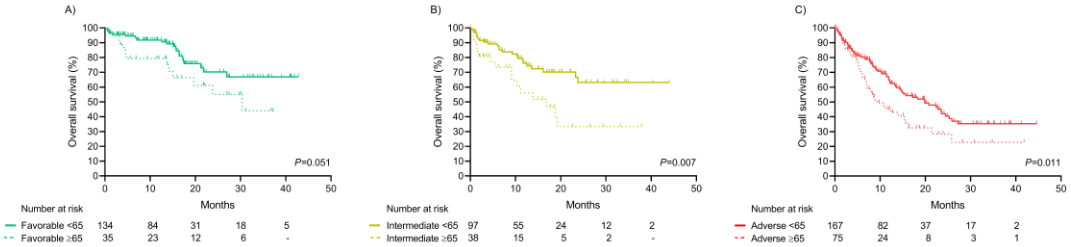

Figure S2. Overall survival assessment between young and elderly AML patients for the 2017 ELN risk categories: A) Favorable, B) Intermediate and C) Adverse.

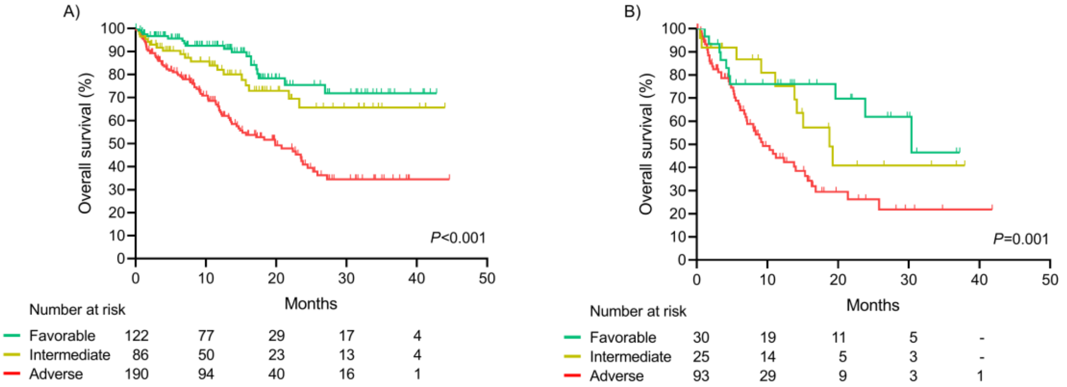

Figure S3. Overall survival after intensive therapy according to 2022 ELN risk for: A) young patients (<65 years) and B) elderly patients (≥65 years)

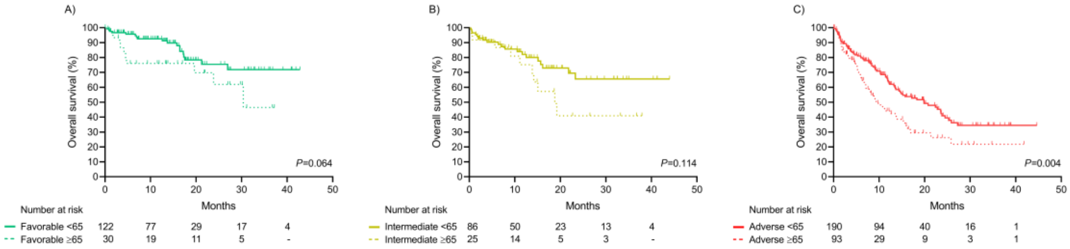

Figure S4. Overall survival assessment between young and elderly AML patients for the 2022 ELN risk categories: A) Favorable, B) Intermediate and C) Adverse.

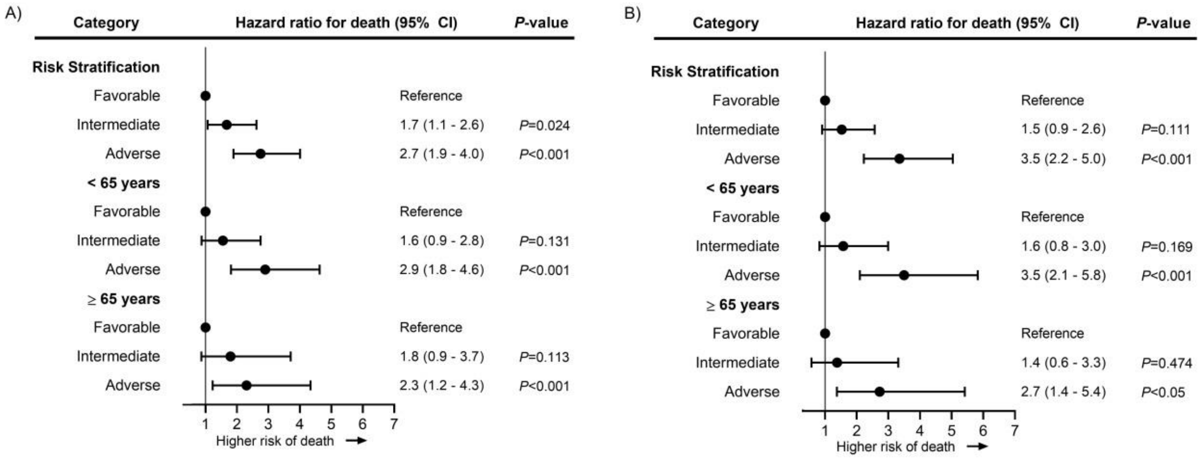

Figure S5. Hazard ratio for A) 2017 ELN and B) 2022 ELN risk categories.

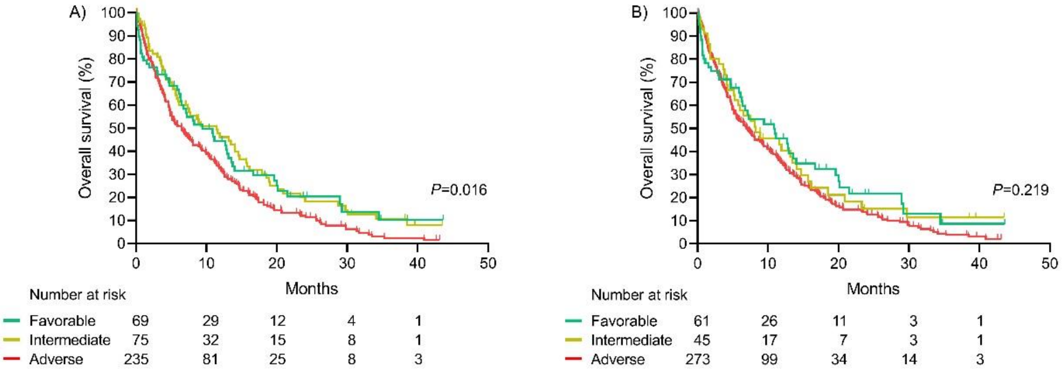

Figure S6. Overall survival analysis in non-intensive treated patients according to: A) 2017 ELN and B) 2022 ELN risk categories.

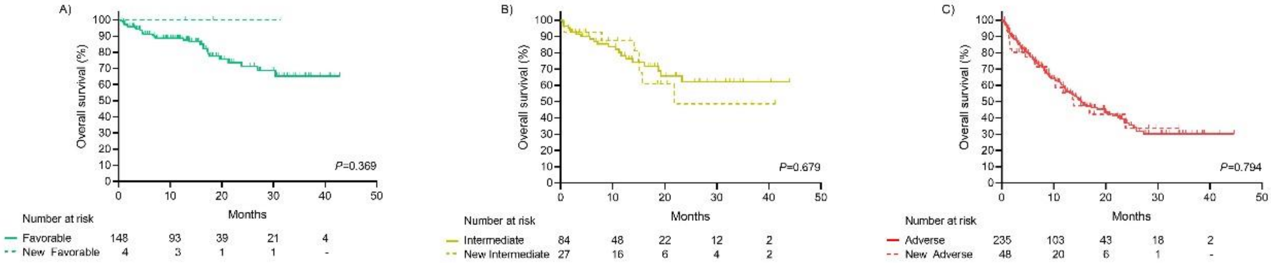

Figure S7. Overall survival analysis of reclassified A) new favorable, B) new intermediate and c) new adverse risk patients according to 2022 ELN and patients who remained in the risk category.

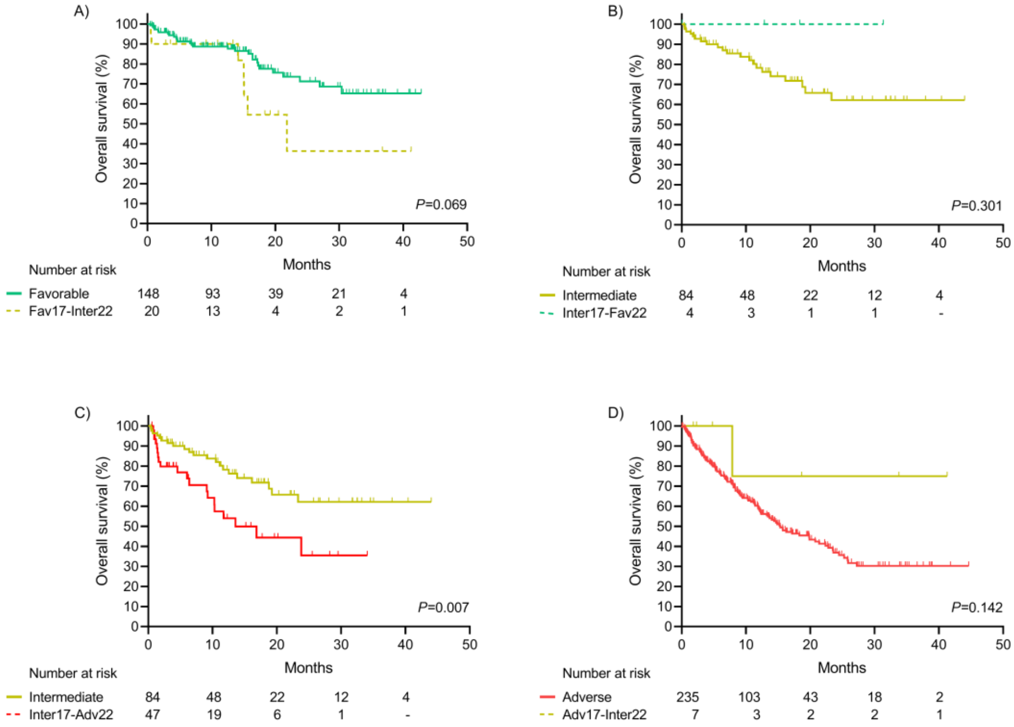

Figure S8. Overall survival analysis of reclassified patients vs. their previous risk group. A) favorable-risk patients vs. new 2022 intermediate-risk. B) intermediate-risk patients vs. new 2022 favorable-risk. C) intermediate-risk patients vs. new 2022 adverse-risk. D) adverse-risk patients vs. new 2022 intermediate-risk. Comparison between favorable-risk patients vs. new 2022 adverse-risk is not shown as only one patient transitioned.

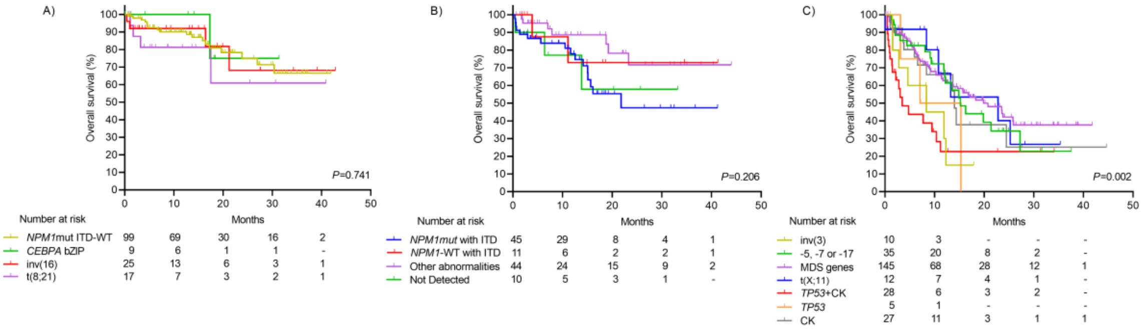

Figure S9. Outcomes of specific genetic subsets with in the 2022 ELN risk categories: A) Favorable, B) Intermediate and C) Adverse. WT: wild-type; CK: Complex karyotype; MDS: myelodysplasia-related genes.

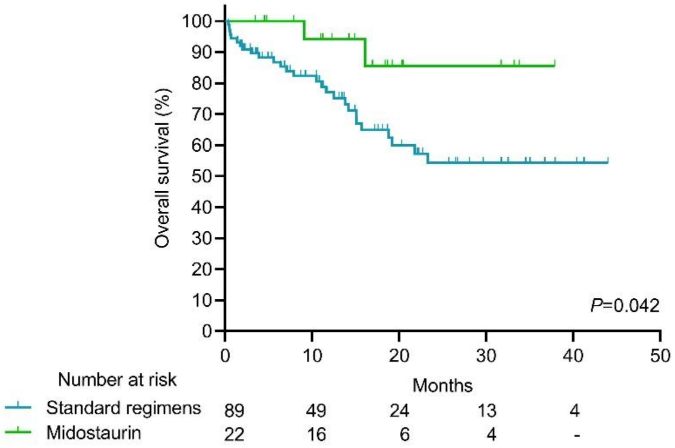

Figure S10. Outcomes from intermediate risk patients with *FLT3*-ITD AML treated with midostaurin inhibitor.

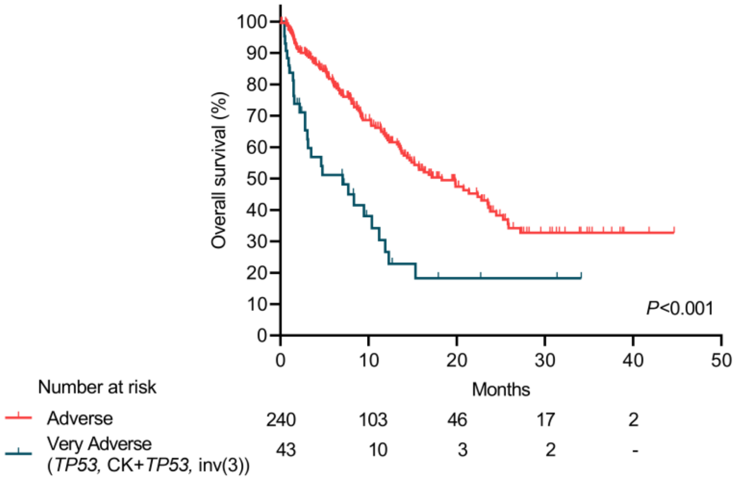

Figure S11. Outcomes from 2022 ELN adverse risk patients and those classified in the proposed as very high-risk.

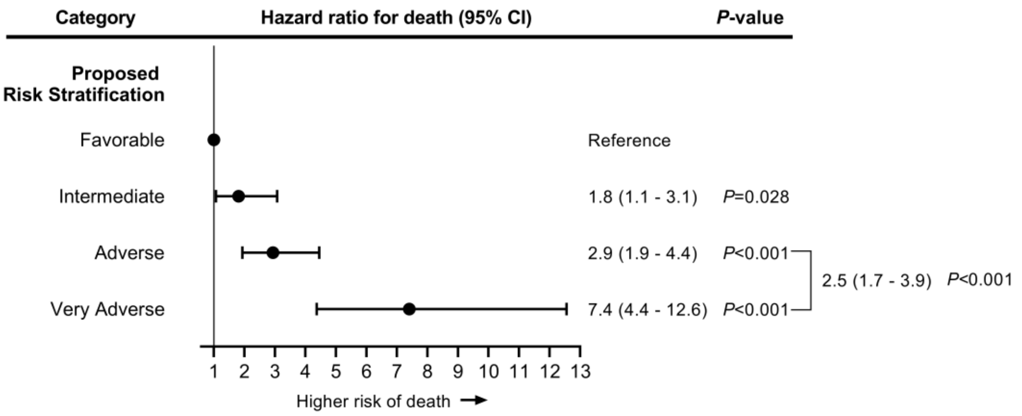

Figure S12. Hazard ratio for the new risk stratification proposal according to the PETHEMA cohort.

Tables

Table S1. Demographic and baseline characteristics of the intensively-treated patients (N=546).

Characteristic	Median	Range	N	(%)
**Age, years**	58.4	18-88	546	100
<65			398	72.9
≥65			148	27.1
**Sex**			546	100
Male			308	56.4
Female			238	43.6
**ECOG**			465	100
0			235	50.5
1			187	40.2
2			31	6.7
3			9	1.9
4			3	0.6
Not available			81	
**WBC (×10^9/L)**	8.7	0.2-374	406	
**BM blast cells, %**	53.0	0-100	463	
**Creatinine, mg/dL**	0.8	0.4-10.3	470	
**MRC cytogenetic risk**			546	100
Favorable			44	8.1
Intermediate			95	17.4
Adverse			134	24.5
Normal karyotype			273	50.0
**AML FAB subtype**			318	100
M0			31	9,7
M1			64	20,1
M2			50	15,7
M4			81	25,5
M5			76	23,9
M6			11	3,5
M7			5	1,6
Not available			228	
**Type of AML**			546	100
De novo			405	74.2
Secondary			141	25.8

Table S2. Patients' characteristics according to 2022 ELN risk group (N=546).

Characteristic	2022 ELN risk categories			P
	Favorable N=152	Intermediate N=111	Adverse N=283	
**Age, years**	53.7 (17.4-75.8)	58.2 (22.9-88.1)	60.9 (18.0-83.9)	<0.001
**Age, group**				
<65	122 (30.7)	86 (21.6)	190 (47.7)	0.006
≥65	30 (20.3)	25 (16.9)	93 (62.8)	
**Sex**				
Male	84 (27.3)	51 (16.6)	173 (56.2)	0,022
Female	68 (28.6)	60 (25.2)	110 (46.2)	
**ECOG**				
0	66 (28.1)	55 (23.4)	114 (48.5)	0,09
1	52 (27.8)	28 (15)	107 (57.2)	
2	10 (32.3)	9 (29)	12 (38.7)	
3	0 (0)	3 (33.3)	6 (66.7)	
4	2 (66.7)	0 (0)	1 (33.3)	
Not available				81
**WBC (×10^9/L)**	20.9 (0.6-339)	16.7 (0.3-374)	5.2 (0.2-314.3)	<0.001
**BM blast cells, %**	58 (20-100)	56.5 (8-100)	48 (4-100)	0.011
**Creatinine, mg/dL**	0.9 (0.4-3.9)	0.8 (0.4-10.3)	0.9 (0.4-7.4)	0.098
**MRC cytogenetic risk**				
Favorable	44 (100)	0 (0)	0 (0)	<0.001
Intermediate	15 (15.8)	24 (25.3)	56 (58.9)	
Unfavorable	0 (0)	3 (2.2)	131 (97.8)	
Normal karyotype	93 (34.1)	84 (30.8)	96 (35.2)	
**AML FAB subtype**				
M0	1 (3.2)	8 (25.8)	22 (71)	<0.001
M1	14 (21.9)	20 (31.3)	30 (46.9)	
M2	21 (42.0)	9 (18.0)	20 (40.0)	
M4	38 (46.9)	18 (22.2)	25 (30.9)	
M5	22 (28.9)	19 (25)	35 (46.1)	
M6	1 (9.1)	1 (9.1)	9 (81.8)	
M7	0 (0)	0 (0)	5 (100)	
Not available				228
**Type of AML**				
De novo	133 (32.8)	99 (24.4)	173 (42.7)	<0.001
Secondary	19 (13.5)	12 (8.5)	110 (78.0)	
**Stem cell transplantation**				
No transplant	44 (22.8)	41 (21.2)	108 (56.0)	<0.001
Autologous	27 (69.2)	7 (17.9)	5 (12.8)	
Allogeneic	21 (14.8)	36 (25.4)	85 (59.9)	
Not available				172

Table S3. Ambiguous patients according to the 2017 ELN risk categories.

N	Karyotype	Genetic abonormalities for ELN-2017 risk categories			Consensus
		**Favorable genetic abnormalities**	**Intermediate genetic abnormalities**	**Adverse genetic abnormalities**	
1	46,XY	Biallelic mutated *CEBPA*	-	Wild-type *NPM1* with *FLT3*-ITD^{high}	Favorable
2	46,XY	-	Mutated *NPM1* with *FLT3*-ITD^{high}	Mutated *RUNX1*	Intermediate

Table S4. Ambiguous patients according to the 2022 ELN risk categories.

N	Karyotype	Genetic abonormalities for 2022 ELN risk categories			Consensus
		**Favorable risk alteration**	**Intermediate risk alteration**	**Adverse risk alteration**	
1	46,XY	*CEBPA* bZIP in-frame	Wild-type *NPM1* with *FLT3*-ITD	-	Favorable
2	46,XX,t(6,9)(p23,q34)	-	Wild-type *NPM1* with *FLT3*-ITD	t(6;9)(p23;q34)	Adverse
3	54,XX,+4,+6,+8,+9,+10,+13,+5,add(16)(q24),+21	-	Wild-type *NPM1* with *FLT3*-ITD	Complex/Monosomal karyotype	Adverse
4	46,XX,t(6,9)(p23,q34)	-	Wild-type *NPM1* with *FLT3*-ITD	t(6;9)(p23;q34)	Adverse
5	46,XY,t(11,19)(q23,p13)	-	Wild-type *NPM1* with *FLT3*-ITD	t(v;11q23.3)	Adverse
6	46,XY,t(3,3)(q21,q26.2)	-	Wild-type *NPM1* with *FLT3*-ITD	t(3;3)(q21;q26.2)	Adverse
7	46,XX,del(7)(q21),del(5)(q31)	-	Wild-type *NPM1* with *FLT3*-ITD	-7, -5 + Mutated MDS-related genes	Adverse
8	47 XY,+8, t(11,19)(q23,p13)	-	Wild-type *NPM1* with *FLT3*-ITD	t(v;11q23.3)	Adverse
9	46,XY,t(6,9)(p22,q34)	-	Wild-type *NPM1* with *FLT3*-ITD	t(6;9)(p23;q34)	Adverse
10	46,XY,t(6,9)(p23,q34)	-	Wild-type *NPM1* with *FLT3*-ITD	t(6;9)(p23;q34)	Adverse

Table S5. Therapeutic approach of the study population: 546 intensively-treated patients and 379 non-intensively treated patients.

Therapeutic Approach	Schedule	N
Intensive chemotherapy	IDA + Ara-C (3+7)	370
	IDA + Ara-C (2+5)	13
	DNR + Ara-C (3+7)	12
	FLAG-IDA (fludarabine + Ara-C + IDA)	22
	IDA + Ara-C (3+7) + Mylotarg	6
	IDA + Ara-C (3+7) + FLT3 Inhibitor	16
	Vyxeos (CPX-351)	30
	Clinical trial	65
	Other intensive chemotherapy	12
		546
Non-intensive therapy	Azacitidine	163
	Decitabine	5
	FLUGA (fludarabine + Ara-C)	142
	FLAGIDA-Lite (flu+LDAC+oral ida)	2
	Low dose ARA-C (LDAC)	4
	Clinical trial	55
	Other non-intensive chemotherapy	8
		379

Appendix A

Institutions and clinicians participating in the PETHEMA epidemiologic registry of acute myeloid leukemia and acute promyelocytic leukemia.

Argentina (Grupo Argentino para el Tratamiento de la Leucemia Aguda - GATLA)— Hospital de Clínicas, Buenos Aires: F. Rojas; H. Longoni; Fundaleu, Buenos Aires: G. Milone, I. Fernández, Clínica Conciencia, Neuquén: R. Ramirez; Hospital Rossi ,La Plata : C. Canepa, S. Saba, G. Balladares, Hospital General San Martin, Parana : G. Milone, C. Ventiurini, R. Mariano, P. Negri; Hospital Italiano de La Plata, La Plata: M. V. Prates, J. Milone; Hospital General San Martín, La Plata:P. Fazio, M. Gelemur; Hospital Clemente Alvarez, Rosario: G. Milone, S. Ciarlo, F. Bezares; Hospital de Córdoba, Córdoba: L. López , Hospital Privado de Córdoba, Córdoba: J. J.García; Instituto Privado Hematología, Paraná: P. Negri, M. Giunta, G. Milone; Hospital Teodoro Alvarez, Buenos Aires: M. Kruss; Hospital Tornú, Buenos Aires: D. Lafalse, G. Milone; Hospital Gobernador Centeno, La Pampa: E. Marquesoni, M. F. Casale; Hospital Italiano de Buenos Aires, Buenos Aires: A. Gimenez , E. B. Brulc, M. A. Perusini; Complejo Médico Policía Federal, La Plata: G. Milone, L. Palmer; Colombia (Asociación Colombian de Hematología y Oncología - ACHO)— Clínica La Estancia, Popayán: M. E. Correa; Fundación Valle del Lili , Cauca: F.J. Jaramillo, J. Rosales; Instituto FOSCAL, Bucaramanga: C. Sossa, J. C. Herrera ; Hospital Pablo Tobón Uribe, Antioquia: M. Arango; Poland (Polish Adult Leukemia Group - PALG)—City Hospital Legnica, Baja Silesia: J. Holojda; IHIT Hematology and transfusiology institute, Warszawa: A. Golos, A. Ejduk; Wojewódzki Szpital Specjalistyczny w Olsztynie, Olsztyn: B. Ochrem; WIM (Military Institute of Medicine in Warsaw), Warszawa: G. Małgorzata; Poland Medical University of Warsaw Banacha, Warszawa:A. Waszczuk-Gajda, J. Drozd-Sokolowska, M. Czemerska, M. Paluszewska; Medical University School Gdansk, Gdansk: E. Zarzycka; Wojewódzki Szpital Specjalistyczny im. Św.. Jadwigi Śląskiej, Opole: A. Masternak; Hospital Brzozow, Brzozow: Dr. Hawrylecka; Medical University Lublin, Lublin: M. Podhoreka, K. Giannopoulos, T. Gromek; Medical University Bialystok, Bialystok: J. Oleksiuk; Silesian Medical University Katowice, Katowice:bA. Armatys, G. Helbig; Universitary Hospital Wroclaw ,Wroclaw: M. Sobas; Poznan University of Medical Sciences, Pozna: A. Szczepaniak; Rydigier City Hospital Krakow, Krakow: E. Rzenno, M. Rodzaj; Collegium Medicum Jagiellonian University Krakow, Krakow: B. Piatkowska-Jakubas; City Hospital Rzeszów,Rzeszów: A. Skret; Medical University Lodz, Lodz: A. Pluta, M. Czemerska; Center of Oncology Kielce, Kielce: E. Barańska; Medical University of Warsaw , Warsaw : M. Paluszewska; Portugal—Hospital de Santa Maria-Lisboa, Lisboa:G. Vasconcelos, J. Brioso; IPOFG Lisboa, Lisboa: A. Nunes, I. Bogalho; Centro Hospitalar e Universitário de Coimbra, Coimbra: A. Espadana, M. Coucelo, S. Marini, J. Azevedo, A. I. Crisostomo, L. Ribeiro, V. Pereira; Centro Hospitalar de Lisboa Central E. P. E, Lisboa: A. Botelho; Instituto Português Oncologia do Porto Francisco Gentil, Porto: J. M. Mariz; Centro Hospitalar São João, Porto: J. E. Guimaraes, E. Aguiar; Centro Hospitalar do

Porto E.P.E. Porto: J. Coutinho; Spain (Programa Español de Tratamiento de las Hemopatías Malignas, PETHEMA)—Complejo Hospitalario Universitario A Coruña, A Coruña: V. Noriega, L. García, C. Varela, G. Debén, M. R. González; Hospital Clínico Universitario de Santiago, A Coruña: M. Encinas, A. Bendaña, S. González, J.L. Bello, M. Albors; Hospital General de Albacete, Albacete: L. Algarra, J.R.Romero, J.S.Bermon, M.J. Varo; Hospital Vinalopó, Alicante: V. López, E. López; Hospital Virgen de los Lirios, Alcoy: C. Mora, C. Amorós; Hospital General Elche, Alicante: E. López, A. Romero; Hospital Torrevieja Salud, Alicante: A. Jaramillo, N. Valdez, I. Molina, A. Fernández, B. Sánchez; Hospital de la Marina Baixa Villajoyosa, Alicante: A. García; Hospital General de Elda, Alicante: V. Castaño, T. López, J. Bernabeu; Hospital de Denia-Marina Salud, Alicante: M.J. Sánchez; Hospital de la Vega Baja de Orihuela, Alicante: C. Fernández; Hospital General de Alicante, Alicante: C. Gil, C. Botella, P. Fernández, M. Pacheco, F. Tarín; J.J. Verdú; Complejo Hospitalario Torrecardenas, Almeria: M.J. García, A. Mellado, M.C. García, J. González; Hospital Central de Asturias, Asturias: T. Bernal, E. Colado, S. Alonso; Complejo Asistencial Ávila, Ávila: I. Recio, M. Cabezudo, J. Davila, M. J. Rodríguez, A. Barez, B. Díaz; Hospital Don Benito-Villanueva, Badajoz: J. Prieto; Institut Catala d'Oncologia L´Hospitalet, Barcelona: M. Arnan, C. Marín, M. Mansilla; Hospital de Cruces, Bizkaia: A. Balaberdi, M. E. Amutio, R. A. del Orbe, I. Ancin, J. C. Ruíz; Hospital Galdakao-Usansolo, Bizkaia: M. Olivalres,C. Gómez, I. gonzález, M. Celis, K. Atutxa, T. Carrascosa, T. Artola, M. Lizuain; Basurtuko Ospitalea, Bizkaia: J .I. Rodriguez, O. Arce, J. A. Márquez, J. Atuch, F. Marco de Lucas, Z. Díez, B. Dávila; Hospital Santos Reyes, Burgos: R. Cantalejo, M. Díaz; Hospital Universitario de Burgos, Burgos: J. Labrador, F. Serra, G. Hermida, F. J. Díaz, P. de Vicente, R. Álvarez: Hospital Santiago Apóstol, Burgos: C. Alonso, Hospital San Pedro de Alcántara, Cáceres: J. M. Bergua; Hospital Campo Arañuelo, Cáceres: N. Ugalde; Hospital Virgen del Puerto, Cáceres: E. Pardal; Hospital General Jerez de la Frontera, Cádiz: R. Saldaña, F. Rodríguez, E. Martín, L. Hermosín; Hospital Universitario Puerta del Mar, Cádiz: M. P. Garrastazul, I. Marchante, J. A. Raposo, F. J. Capote; Hospital U. Marqués de Valdecilla, Cantabria: M. Colorado, A. Batlle, L. Yañez, S. García, P. González, E. M. Ocio, M. Briz, A. Bermúdez, S. García; Consorcio Hospitalario Provincial de Castellón, Castellón: C. Jiménez, S. Beltrán; Hospital de Vinaroz: M. Montagud; Hospital Universitario de La Plana, Castellón: I. Castillo; Hospital General de Castellón, Castellon: R. García, A. Gascón, J. Clavel, A. Lancharro, L. Lnares; Hospital Santa Bárbara , Ciudad Real: M. M. Herráez, A. Milena; Hospital Virgen de Altagracia, Ciudad Real: M. J. Romero, Hospital General de Ciudad Real, Ciudad Real: B. Hernández, C. Calle, R. Benegas; Hospital Gutierrez Ortega de Valdepeñas, Ciudad Real: Dr. Bolívar; Hospital General La Mancha Centro, Ciudad Real: M. A. Pozas; Hospital Reina Sofia, Córdoba: J. Serrano, F. J. Dorado, J. Sánchez, M. C. Martínez, E. Prados; Hospital Virgen de la Luz, Cuenca: C. J. Cerveró, M. J. Busto; Hospitales HUVN-HC San Cecilio de Ganada, Granada: M. Bernal, E. López, L. Moratalla, Z. Mesa, M. Jurado, A. Romero, P. González; Complejo Hospitalario Universitario Granada, Granada: L. Moratalla, A. Romero, L. López;

Hospital Universitario de Guadalajara, Guadalajara: M. Díaz, D. De Miguel, A. B. Santos, J. Arbeteta; Hospital Donostia, Donosti: E. Pérez, N. Caminos, N. Uresandi, N. Argoitiaituart, T. Artola, J. Swen, A. Uranga, I. Olazaba, M. Lizuain, E. Gainza, P. Romero; Hospital Juan Ramón Jimenez Huelva, Huelva: E. Gil, A. J. Palma, K. G. Gómez, M. Solé, J. N. Rodríguez; Hospital San Jorge, Huesca: I. M. Murillo, J. Marco, J. Serena, V. Marco; Hospital de Barbastro, Huesca: M. Perella, L. Costilla; Hospital General Ciudad de Jaen, Jaén: J. A. López, A. Baena, P. Almagro; Hospital San Pedro de Logroño, La Rioja: M. Hermosilla, A. Esteban, B. A. Campeny, M. J. Nájera, P. Herrra; Hospital Insular de Las Palmas, Las Palmas: R. Fernández, J. D. González, L. Torres; Hospital Dr. Negrín, Las Palmas: S. Jiménez; M. T. Gómez, C. Bilbao, C. Rodríguez; Hospital Doctor José Molina Orosa, Las Palmas: A. Hong, Y. Ramos de Laón, V. Afonso; Hospital Universitario de León, León: F. Ramos, M. Fuertes; Hospital Comarcal del Bierzo, León: E. de Cabo, C. Aguilera, M. Megido; Hospital Universitari Arnau de Vilanova de Lleida, Leida: T. García; Hospital Lucus Augusti, Lugo: E. Lavilla, M. Varela, S. Ferrero, M. J. Sánchez, L. López, J. Arias, L. Vizcaya; Hospital Infanta Sofía, Madrid: A. Roldán, A. Vilches, M. J. Penalva, J. Vázquez; Hospital Central de la Defensa Gómez Ulla, Madrid: M. T. Calderón, A. Matilla, C. Serí, M. J. Otero, N. García, E. Sandoval; Hospital de Fuenlabrada, Madrid: C. Franco, R. Flores, P. Bravo, A. López; Hospital Fundación Jiménez Díaz, Madrid: J. L. López, C. Blas, A. Díez, J. M. Alonso, C. Soto, A. Arenas; Hospital U. Príncipe de Asturias, Madrid: J. García, Y. Martín, P. S. Villafuerte, E. Magro; Hospital Puerta de Hierro, Madrid: G. Bautista; A. De Laiglesia; Hospital Gregorio Marañón, Madrid: G. Rodríguez, L. Solán, M. Chicano, P. Balsalobre, S. Monsalvo, P. Font, D. Carbonell, C. Martínez; Hospital U. La Paz, Madrid: K. Humala, A. E. Kerguelen, D. Hernández, M. Gasior, P. Gómez, I. Sánchez; Hospital Madrid Norte Sanchinarro, Madrid: S. Redondo, L. Llorente, M. Bengochea, J. Pérez; Hospital Sanitas Torrejón, Madrid: A. Sebrango, M. santero, A. Morales; Hospital La Princesa, Madrid: A. Figuera, P. Villafuerte, A. Alegre, E. Fernández; Hospital Ruber Internacional, Madrid: A. Alonso; Hospital 12 de Octubre, Madrid: M. P. Martínez, J. Martínez, M. T. Cedena, L. Moreno, R. Ayala; MD Anderson Cancer Center, Madrid: A. De la Fuente; Hospital Sanitas La Zarzuela, Madrid: D. García; Hospital Universitario Quiron, Madrid: C. Chamorro, V. Pradillo, E. Martí, J. M. Sánchez, I. Delgado, A. Alonso; Hospital Rey Juan Carlos, Madrid: B. Rosado, A. Velasco, C. Miranda, G. Salvatierra, J. M. Alonso,, J. L. López; Hospital Infanta Leonor, Madrid: M. Foncillas, J. A. Hernández; Hospital Universitario de Getafe, Madrid: C. Escolano, L. García, I. Delgado; Hospital Clínico San Carlos, Madrid: C. Benabente, R. Martínez, M. Polo, E. Anguita; Hospital Universitario Severo Ochoa, Madrid: R. Riaza, G. Amores, M. J. Requena; Hospital Universitario Fundación Alcorcón, Madrid: F. Javier, L. Villaloón; Hospital Universitario Moncloa, Madrid: C. Aláez, V. Pradillo, S. Nistal, B. Navas; Hospital Universitario de Móstoles, Madrid: J. Sánchez, M. A. Andreu; Hospital Ramon y Cajal, Madrid: P. Herrera, J. López; Hospital U. Virgen de la Victoria, Málaga: M. García, M. J. Moreno, A. Fernández, M. P. Queipo; Hospital Quirónsalud Málaga, Málaga: A. Hernández;

Hospital Regional de Málaga, Málaga: M. Barrios, A. Heiniger, A. Jiménez, A. Contento, F. López, M. Alcalá; Hospital Vithas Xanit Internacional, Málaga: S. Lorente, M. González, E. M. Morales, J. Gutierrez; Hospital Virgen del Castillo, Murcia: M. J. Serna, V. Beltrán; Hospital Santa Lucía de Cartagena, Murcia: M. Romera, M. Berenguer, A. Martínez, A. Tejedor; Hospital Morales Meseguer, Murcia: M. L. Amigo, F. Ortuño, L. García, A. Jerez, O. López; Hospital U. Virgen de la Arrixaca, Murcia: J. M. Moraleda, P. Rosique, J. Gómez, M. C. Garay; Hospital Los Arcos Mar Menor, Murcia: P. Cerezuela, C. Martínez, A. B. MArtínez, A. González; Hospital STª Mª del Rosell, Murcia: J. Ibáñez; Clínica San Miguel, Navarra: M. J. Alfaro; Complejo Hospitalario de Navarra, Navarra: M. Mateos, M. A. Goñi, M. A. Araiz, A. Gorosquieta, M. Zudaire, M. Viguria, A. Zabala, M. Alvarellos, I. Quispe, M. P. Sánchez, G. Hurtado, M. Pérez, Y. Burguete, N. Areizaga, T. Galicia; Clínica Universitaria de Navarra, Navarra: J. Rifón, A. Alfonso, F. Prósper,M. Marcos, L. E. Tamariz, V. Riego. A. Manubens, M. J. Larrayoz, M. J. Calasanz, A. Mañú, B. Paiva, I. Vázquez, L. Burgos; Complejo Hospitalario de Ourense (CHOU), Ourense: M. Pereiro, M. Rodríguez, M. C. Pastoriza, J. A. Mendez, J. L. Sastre, M. Iglesias, C. Ulibarrena, F. Campoy; Hospital Valdeorras, Ourense: D. Jaimes; Hospital Rio Carrión, Palencia: J. M. Alonso, B. Albarrán, J. Solano, A. Silvestre; Complexo Hospitalario Universitario de Vigo, Vigo: C. Albo, S. Suarez, C. Loureiro, I. Figueroa, M. Rodríguez, M. A. Fernández, A. Martínez, C. Poderós, J. Vazquez, L. Iglesias, A. Nieto, T. Torrado, A. M. Martínez; Hospital Provincial de Pontevedra, Pontevedra: M.L. Amador, P. Oubiña, E. Feijó, A. Dios, I. Loyola, R. Roreno; Hospital POVISA, Pontevedra: A. Simiele, L. Álvarez, V. Turcu; Hospital U. Salamanca, Salamanca: B. Vidriales, M. González, R. García, A. Avendaño, C. Chillón, E. Pérez, V. González; Hospital General La Palma, Santa Cruz de Tenerife: J. V. Govantes, S. Rubio, M. Tapia; Hospital General de Segovia, Segovia: C. Olivier, J. A. Queizán; Hospital U. Virgen Macarena, Sevilla: : O. Pérez, J. A. Vera, C. Muñoz, A. rodriguez, N. González; Hospital U. Virgen del Rocio, Sevilla: J. A. Pérez, E. Soria, I.Espigado, J. Falantes, I. Montero, P. García, E. Rodríguez, E. Carrillo, T. Caballero, C. García; Hospital Virgen de Valme, Sevilla: C. Couto, I. Simón, M. Gómez; Hospital Virgen del Mirón de Soria, Soria: C. Aguilar; Hospital Universitario Canarias, Tenerife: B. J. González, S. Lakhwani, A. Bienert, B. González; Hospital Universitario Nuestra Señora de Candelaria, Tenrife: A. Cabello, A. Y. Oliva, H. González; Hospital Obispo Polanco, Teruel: N. González, Hospital de Alcañiz, Teruel: L. Sancho, M. Paricio, L. Perdiguer; Hospital General Nuestra Señora del Prado, Toledo: F. Solano, A. Lerma, M. D. Martínez; Hospital Virgen de la Salud de Toledo, Toledo: M. I. Gómez, A. Yeguas; Hospital U. La Fe, Valencia: P. Montesinos, E. Barragán, C. Sargas, R. Amigo, D. Martinez, B. Boluda, R. Rodríguez, E. Acuña, I. Cano; Hospital de Requena, Valencia: A. Escrivá, M. Pedreño; Hospital de Lluis Alcanyis de Xativa, Valencia: R. Renart; IVO (Instituto Valenciano de Oncología), Valencia: A. Navalón; Hospital de Sagunto, Valencia: I. Castillo, M. Orts; Hospital Dr. Peset, Valencia: M. J. Sayas, M. J. Fernández, M. L. Juan, E. Gómez, M. Gimeno, E. Donato, M. Cejalvo, J. Marco; Hospital Clínico Universitario, Valencia: M.

Tormo, M. Calabuig, B. Navarro, I. Martin, E. Villamont, A. Miralles; Hospital de La Ribera, Valencia: R. Lluch; Hospital Casa de la Salud, Valencia: J. García; Hospital de Gandía, Valencia: M. Moragues, M. A. Ruiz; Hospital Arnau de Vilanova, Valencia: A. López, C. Benet, M. Valero; Hospital General de Valencia, Valencia: M. Linares, R. Collado, M. Orero, P. Ibañez, M. J. Lis, P. L. Pérez, M. Roig, M. López, A. V. Mena; Hospital Manises, Valencia: I. Picón, V. Cánovas, A. Palacios, E. Martí; Hospital Clínico de Valladolid, Valladolid: R. Cuello, J. Borrego, M. burgois; Hospital Rio Hortega, Valladolid: A. Cantalapiedra, O. Norberto, E. Angomas, B. Cidoncha; Hospital Universitario Araba, Victoria: L. Cuevas, D. Robles, A. Mendiazabal, I. Oiartzabal, J. M. Guinea de Castro; Hospital Virgen de la Concha, Zamora: C. Montes, M. Pérez, L. García; Hospital Royo Villanova, Zaragoza: V. Carrasco, A. Pérez, L. López, J. J. Moneva; Hospital Clínico U. Lozano Blesa, Zaragoza: M. Olave, E. Bonafonte, L. Mayor, G. Azaceta, L. Palomera; Hospital Ernest Lluch Martin, Zaragoza: M. Malo, M. J. Escobar; Hospital Quiron Salud Zaragoza, Zaragoza: J. M. Grasa; Hospital Miguel Servet, Zaragoza: B. De Rueda, A. Aulés, C. Salvador, V. Ansó, A. Iborra, P. Delagado, A. Rubio; Uruguay —Hospital de Clínicas, Montevideo: M. Stevenazzi, I. Alpire, V. Irigoin, L. Díaz, C. Guillermo, R. Guadagna, S. Grille, C. Oliver, M. Boada, V. Vales; Hospital Maciel, Montevideo: A. I. Prado; COMERO (Cooperativa Medica de Rocha), Rocha: A. P. De los Santos.
